# SR31747A is a sigma receptor ligand exhibiting antitumoural activity both *in vitro* and *in vivo*

**DOI:** 10.1038/sj.bjc.6600709

**Published:** 2003-02-10

**Authors:** Y Berthois, B Bourrié, S Galiègue, H Vidal, P Carayon, P M Martin, P Casellas

**Affiliations:** 1 Laboratoire de cancérologie expérimentale EA2671, IFR Jean Roche, Faculté de Médecine Secteur Nord, Boulevard Pierre Dramard, 13916 Marseille cedex 20, France; 2 Département Immunologie-Oncologie, Sanofi Synthelabo, 371 rue du Professeur Joseph Blayac, 34184 Montpellier cedex 04, France

**Keywords:** SR31747A, sigma receptors, human sterol isomerase, proliferation inhibition, breast and prostate cancer

## Abstract

SR31747A is a recently described sigma receptor ligand that binds SR31747A-binding protein 1 (SR-BP) and emopamil-binding protein (EBP) (also called the sigma1 receptor and the human sterol isomerase (HSI), respectively), and has immunoregulatory and antiproliferative activities. To further investigate its antitumour activity and focusing on cancers, which are sensitive to the molecule, we measured the proliferation of different human epithelial breast or prostate cancer cell lines following *in vitro* and *in vivo* SR31747A treatment. Firstly, *in vitro*, we found that nanomolar concentrations of SR31747A dramatically inhibited cell proliferation in both hormono-responsive and -unresponsive cancer cell lines. Secondly, tumour development was significantly decreased in mice treated with SR31747A. In an attempt to decipher the SR31747A mode of action, we found that the two binding sites may not fully account for this activity. Indeed, while competitive experiments indicated that EBP prevails in mediating SR31747A antiproliferative activity, an analysis of the expression of both receptors indicated that the cellular sensitivity to SR31747A is not correlated with either EBP or SR-BP expression. These data suggest that additional binding sites may exist. Preliminary binding studies demonstrated that SR31747A also binds to sigma2, a protein that has not yet been cloned, but which is considered as a potential marker of the proliferative status of tumour cells. Altogether, our data demonstrate the antitumoural activity of SR31747A both *in vitro* and *in vivo* in two different cancer models, broaden the spectrum of its binding proteins and enhance the potential for further therapeutic development of the molecule.

SR31747A is a selective peripheral sigma binding site ligand whose biological activities include immunoregulation and inhibition of cell proliferation. For instance, SR31747A was shown to modulate dramatically proinflammatory and anti-inflammatory cytokine responses by stimulating SEB (staphylococcal enterotoxin B)- or LPS (lipopolysaccharide)-induced serum release of IL-10, while also inhibiting the systemic release of IL-2, IL-4, GM-CSF, IL-6 and TNF*α* (Derocq *et al*, 1995; Bourrie *et al*, 1996). The antiproliferative effect of nanomolar SR31747A concentrations was demonstrated *in vitro* on a series of various established animal cell lines (Labit-Le Bouteiller *et al*, 1998). In humans, SR31747A is known to bind two proteins with nanomolar affinity. They include SR31747A-binding protein 1 (SR-BP) and the human sterol isomerase (HIS), also called EBP for emopamil-binding protein. SR-BP is a 28 kDa protein identical to the sigma1 receptor (Kekuda *et al*, 1996; Jbilo *et al*, 1997). Homology sequence studies indicated that SR-BP is related to the fungal C8–C7 sterol isomerase encoded by the ERG2 gene (Silve *et al*, 1996). Both proteins are 30% homologous. Contrasting with SR-BP, which does not show any enzyme activity and does not complement the ERG2 gene defect in yeast, EBP is the human sterol isomerase counterpart of the yeast ERG2 protein (Hanner *et al*, 1995; Silve *et al*, 1996). In humans, the enzyme is a 26 kDa, protein, which belongs to the sterol biosynthesis pathway and catalyses the conversion of 5*α*-cholesta-8,24-dien-3*β*-ol (zymosterol) and 5*α*-cholesta-8-en-3*β*-ol (zymostenol, Δ8-cholestenol) to their corresponding Δ7-isomers. In the yeast *Saccharomyces cerevisiae*, SR31747A is known to bind ERG2 with nanomolar affinity and to arrest yeast cell proliferation through ERG2 inhibition (Silve *et al*, 1996; Jbilo *et al*, 1997). In mammals, even though two different cellular targets for SR31747A have been identified, the molecule mode of action is not yet fully understood. Some lines of evidence suggest that the two receptors play a different role in mediating SR31747A effects. *In vitro* pharmacological analyses using an SR-BP-specific ligand indicated that SR31747A-induced inhibition of T-cell proliferation was quite consistent with an SR-BP receptor-mediated event (Jbilo *et al*, 1997), suggesting that the immunoregulatory properties of SR31747A are predominantly SR-BP dependent. By contrast, preceding results suggested that the antiproliferative effects of the SR31747A are mediated by inhibition of cholesterol biosyn-thesis through the blockade of EBP. Proliferation of a series of established animal breast, testis and kidney cell lines was shown to be dramatically altered by SR31747A with a concomitant accumulation of the Δ8-cholesterol isomer (Labit-Le Bouteiller *et al*, 1998). Cell growth processes are strictly linked to the cholesterol biosynthetic pathway, which is a multistep reaction. The cholesterol needed for new membranes and farnesylation of ras and G proteins may be derived from endogenous synthesis or exogenous sources, principally low-density lipoproteins (LDL). As the inhibition of cholesterol biosynthesis has been shown to reduce significantly tumour cell growth, this pathway constitutes a highly promising target for anticancer drug design (Rao *et al*, 1995). In this context, SR31747A could be an important potential antitumour agent. To further investigate the SR31747A antiproliferative effect, we first addressed its potential antitumoural activity both *in vitro* and *in vivo* by measuring the proliferative activity of different breast and prostate cancer cell lines and the corresponding tumour development in the mouse xenograft model following SR31747A treatment. Both hormono-dependent and -independent cells have been tested in order to analyze whether there was a correlation between the hormonal status and SR31747A efficacy. In addition, we analysed the role of each receptor in mediating SR31747A antiproliferative activity using a pharmacological approach and by investigating potential correlations between EBP or SR-BP expression and the cellular sensitivity to the drug.

## MATERIALS AND METHODS

### Reagents

SR31747A, anti-EBP and anti-SR-BP antibodies (Jbilo *et al*, 1997; Dussossoy *et al*, 1999) were produced and provided by Sanofi Synthélabo Research Laboratories (Montpellier, France). The rabbit anti-EBP polyclonal antibody was targeted against the N-terminal (2–25) peptide of the human sterol isomerase (Dussossoy *et al*, 1999). The anti-SR-BP antibody was a mouse monoclonal antibody raised against the human purified SR-BP, also called sigma1 receptors (Jbilo *et al*, 1997). Estradiol (E2), tamoxifen, 4-hydroxytamoxifen (OH-TAM), testosterone (TEST) and flutamide were from Sigma (France). (+) Pentazocine was supplied by Research Biochemicals Inc. (Natick, MA, USA).

### Cell lines and culture conditions

Hormono-responsive MCF-7 cells from a breast adenocarcinoma pleural effusion (Soule *et al*, 1973) were obtained from Dr M Lipman's laboratory (NIH, Bethesda, MD, USA) and cultured in 50% Dulbecco's minimum essential medium/50% Ham's F12 (DMEM/F12, 1 : 1, v v^−1^) supplemented with 16 ng ml^−1^ insulin, 2 mM L-glutamine, 10 mM Hepes buffer, 50 UI ml^−1^ penicillin, 50 *μ*g ml^−1^ streptomycin and 10% heat-inactivated fetal calf serum (FBS). Another MCF-7 cell line, MCF-7AZ was obtained from Dr M Mareel (Belgium) at passage 360 and was grown in the same medium as that of MCF-7 cells. MCF-7/LCC1 cells were derived from MCF-7 cell lines and obtained from Dr M Lippman (V Lombardi Cancer Center, Georgetown). The MCF-7/LCC1 variant is hormono-independent (Thompson *et al*, 1993). MCF-7/LCC1 cells were cultured in phenol red-free DMEM/F12 (1 : 1, v v^−1^) containing 5% steroid-stripped FBS (DCC). The MCF-7 variant MCF-7LY2 was also provided by Dr M Lipman. This variant cell line, selected *in vitro* for its resistance to the growth-inhibitory effects of the antioestrogen LY117018 (Davidson *et al*, 1986), was maintained in DMEM/F12 (1 : 1, v v^−1^) supplemented with 10% FBS. The hormono-unresponsive MDA-MB-321 (Cailleau *et al*, 1974) and BT-20 (Lasfargues and Ozzello, 1968) cell lines were also established from a metastatic human breast cancer tumour. MDA-MB-231 cells were maintained in Leibovitz L15 culture medium supplemented with 10 mM Hepes buffer, 6 *μ*g ml^−1^ human insulin, 2 mM L-glutamine, 1% nonessential amino acids, 50 UI ml^−1^ penicillin, 50 *μ*g ml^−1^ streptomycin and 10% FBS. BT20 cells were maintained in an RPMI medium containing 3 *μ*g ml^−1^ human insulin, 2 mM L-glutamine, 50 UI ml^−1^ penicillin, 50 *μ*g ml^−1^ streptomycin and 15% FBS.

The hormono-responsive prostate cancer cell line LNCaP was originally derived from a patient with metastatic prostate cancer of pelvic lymph nodes. The hormono-unresponsive DU145 and PC3 cell lines were, respectively, established from brain and bone-marrow metastase prostatic carcinoma. The three prostatic cell lines were obtained from the American Type Culture Collection (Rockville, MD, USA) and cultured in RPMI 1640 medium supplemented with 10% FBS.

All cells were cultured at 37°C in a humid atmosphere of 5% CO_2_ in air, except for the MDA-MB-231 cell line that was cultured in the absence of CO_2_.

### Primary culture of normal breast epithelial cells (NBECs)

Epithelial cells were grown from tissue specimens obtained, after informed consent, from nine women between 16 and 50 years of age who had undergone reduction mammoplasty for cosmetic reasons. Normal state of breast tissues was controlled by histopathological examination. After mechanical dissociation with scissors, tissues were incubated at 37°C with constant shaking in a medium containing 150 U ml^−1^ hyaluronidase (Sigma-Aldrich, France) and 250–500 U ml^−1^ collagenase (Sigma-Aldrich, France). Digestion was monitored under an inverted microscope. Released organoids were isolated by density gradient centrifugation for 30 min at 800 g, using 1.077 g ml^−1^ lymphocyte separation medium. Organoids were then plated in a DMEM/F12 (1 : 1, v v^−1^) medium containing 5% FBS, 10 *μ*g ml^−1^ insulin, 5×10^–6^ M cortisol (Sigma-Aldrich, France), 2 ng ml^−1^ EGF (Euromedex, France), 100 ng ml^−1^ cholera toxin (Sigma-Aldrich, France), 2 mM L-glutamine, 50 U ml^−1^ penicillin and 50 mg ml^−1^ streptomycin (B1 medium). Cells were maintained at 37°C in a humid atmosphere of 5% CO_2_ in air. When cells attained preconfluence, the medium was replaced by B2 medium, which differed from B1 medium by its lower CaCl_2_ concentration (0.060 *vs* 1.05 mM). Under these conditions, cells released floating daughter cells that were collected and replaced in the B1 medium. The epithelial phenotype of the cultured cells was verified by immunocytochemistry of specific markers, as previously described (Mosmann, 1983).

### Cell growth experiments

Cell growth was determined in six- or 24-well culture plates. Cells were seeded in their respective culture media at densities that varied as a function of their proliferation rate. Cells were allowed to grow for 2 days and then treated for 5–6 days with different SR31747A concentrations. Treatments for breast cancer cell lines were performed in 0.1% FBS, and 1% FBS for prostatic cancer cell lines. Media were renewed every 2 days. At the end of treatment, cells were harvested and the cell number was determined using a cell counter. Cell proliferation was also evaluated by a colorimetric method based on the cellular conversion of tetrazolium salt (MTT) into a blue formazan product (Feulgen and Rossenbech, 1924). The SR31747A antiproliferative effect was compared with that of OH-TAM in 0.1% FBS or flutamide in 1% FBS. To study SR31747A route of action, either 10^–5^ and 10^–6^ M (+) pentazocine or 1, 2, 10 and 20 *μ*g ml^−1^ cholesterol was added to 0.1% FBS when analysing breast cancer cell line responses (MCF7 and MDA-MB-231) or 1% when analysing prostate cancer cell line responses (DU145 and LnCaP), treatment lasted 4 days. These compounds were used to analyse specifically the route of action of SR31747A, as they make it possible to discriminate between EBP and SR-BP. Pentazocine is a specific SR-BP ligand, while the use of cholesterol reverses the blockade of EBP.

### Determination of EBP and SR-BP expression levels by flow cytometry

The analysis was performed with the cell lines used in the *in vitro* antiproliferative test. Normal cells included were lymphocytes and monocytes. After growth in the medium containing 0.1 or 1% serum for breast or prostate cell lines, respectively, 10^6^ cells were fixed overnight at room temperature in 500 *μ*l of 1% paraformaldehyde in PBS. After washes in PBS, cells were permeabilised in PBS containing 1% BSA and 0.1% saponin. Expression of both proteins was determined according to Vogt *et al*, (1989). The EBP antigen number was determined by direct immunofluorescence using an FITC-conjugated rabbit polyclonal anti-EBP antibody and standard FITC-conjugated beads (DAKO). The SR-BP expression level was measured by indirect immunofluorescence assay using 1 *μ*g murine monoclonal anti-SR-BP antibody and FITC-conjugated anti-mouse immunoglobulins (DAKO). The SR-BP antigen number was calculated using a quantitative immunofluorescence assay kit, according to the manufacturer's instructions (DAKO). Experiments were analysed with a FACScan flow cytometer (Becton-Dickinson, France).

### *In vivo* studies

The present animal experiments complied with the European and French laws and with the guiding principles for experimental procedures as set forth in the Declaration of Helsinki and are in accordance with the UK Guidelines for the Welfare of Animals in Experimental Neoplasia (UKCCCR, 1998). Homozygous female athymic nude mice (nu+/nu+, balb/c strain) were obtained from Iffa-Credo (L'Arbresle, France). Animals were housed and maintained in pathogen-limited conditions under filtered laminar air flow hoods at 22–25°C with a 12 h light : 12 h darkness photoperiod. Sterilised food and water were provided *ad libitum*. Six-week-old mice were used to initiate the experiments. Cells from preconfluent monolayers of tumoural cell lines were harvested, then washed and resuspended in PBS supplemented with 1 mM CaCl_2_. 1–5×10^6^ cells in 200 *μ*l of cell suspension were inoculated s.c. in both flanks of animals through a 25-gauge needle. Animal treatments were initiated the following day. All compounds were dissolved in ethanol, Tween 80 and 0.9% NaCl (1 : 1 : 20, v v^−1^). Control animals (10 mice) received i.p. the vehicle, whereas the animals of the studied groups (10 mice per group) were given i.p. daily doses of SR31747A (25 mg kg^−1^), tamoxifen (1 mg kg^−1^), estradiol (100 *μ*g kg^−1^), testosterone (100 *μ*g kg^−1^) or flutamide (2.5 mg kg^−1^), alone or in combination as indicated. A dose of 25 mg SR31747A, once a day, was chosen for *in vivo* studies as this is highly sufficient to maintain an effective plasma level and to saturate all the binding sites (data not shown). Tumours were measured with Vernier calipers at different times. Two perpendicular diameters were recorded, and the tumour volume was calculated according to the formula: (width)^2^/2×length/2. After 2–3 months of treatment, the animals were killed by cervical dislocation and tumours were removed post mortem, weighed and stored in liquid nitrogen. Values relative to tumour size are the mean values obtained for each experimental group (total tumour load divided by the number of tumours). For comparative purposes, tumour sizes or weights in each group are reported in Results as a percentage of the size or weight of the tumour observed in the control group at the end of treatment. Experiments were performed twice.

### Statistical analysis

All values are expressed as mean±s.d. of several determinations. The Student's *t*-test was used for comparison of the experimental data. *, *P*<0.05; **, *P*<0.01.

## RESULTS

### Effect of SR31747A on the proliferation of human breast cancer cell lines 

**Figure 1:**
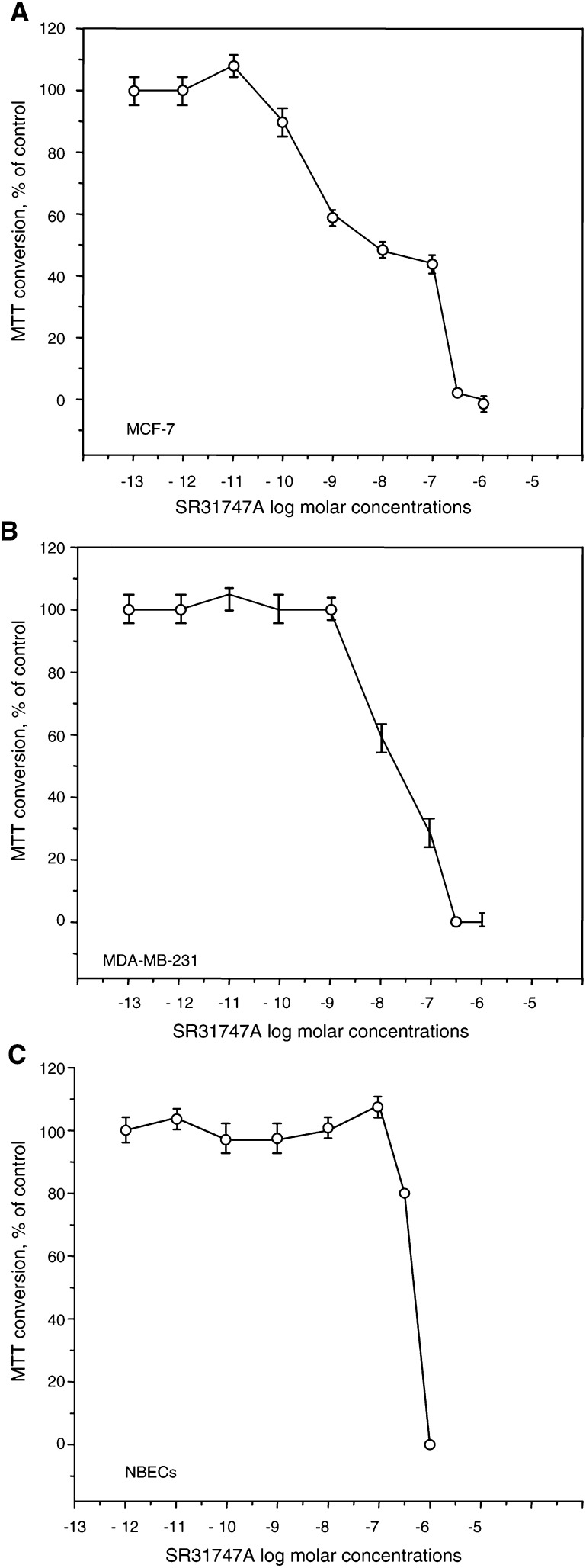
Antiproliferative effect of SR31747A on different breast cancer epithelial cell lines and on NBECs in primary culture. Treatments with different SR31747A concentrations were performed in 0.1% FBS, as described in Materials and Methods. Representative curves are shown. effects on hormono-responsive MCF-7 cells (**A**), hormono-unresponsive MDA MB 231 cells (**B**), or NBECs (**C**). The results are expressed as a percentage of MTT conversion measured in untreated cells.

The effect of SR31747A on cell proliferation was first evaluated *in vitro* on a series of human breast epithelial cancer cell lines. Representative data are reported in Figure 1A

, B and demonstrate that SR31747A clearly induced concentration-dependent inhibition of cell proliferation, irrespective of the cell line considered, that is either hormono-responsive (Figure 1A) or -unresponsive (Figure 1B) cells. The antiproliferative potency of SR31747A varied with the cell line considered and showed an IC_50_ ranging from 10^–10^ to 10^–8^ M (Table 1

**Table 1:** Effect of SR31747A and either OH-TAM or Flutamide on the proliferation of breast cancer epithelial cell lines, NBECs and prostatic cancer epithelial cell lines

	**IC_50_, molar concentrations**	
**Breast cancer cell lines**	**SR 31747A**	**OH-TAM**
MCF-7	2×10^−9^–10^−8^	10^−11^
MCF7-7AZ	10^−9^–10^−8^	3×10^−11^
MCF-7LCC1	10^−9^	5×10^−7^
MCF-7LY2	5×10^−11^–10^−10^	5×10^−7^
MDA-MB-231	10^−8^–5×10^−8^	3×10^−6^
BT20	10^−9^	10^−6^
NBECs	5×10^−7^	10^−8^
		
Prostate cancer cell lines	SR 31747A	Flutamide
LNCaP	10^−8^	10^−6^
DU145	10^−8^	10^−6^
PC3	10^−7^	10^−6^

). While most of the MCF-7-derived cell lines exhibited an IC_50_ of 10^–9^–10^–8^ M for SR31747A, MCF-7LY2 cells appeared to be the most sensitive cells since 50% cell growth inhibition was observed with 10^–11^–10^–10^ M SR31747A. The proliferation of the hormono-independent MDA-MB231 and BT-20 cells was similarly affected by SR31747A, with an IC_50_ of 10^–8^ and 10^–9^ M, respectively (Table 1). For comparative purposes, we tested the effect of the antioestrogen OH-TAM on cell proliferation. As reported in Table 1, the OH-TAM, which also blocks EBP activity, appeared to be more efficient than SR31747A at inhibiting the proliferation of the hormono-sensitive MCF-7 and MCF-7AZ cell lines. By contrast, the proliferation of the hormono-independent MDA-MB231 and BT-20 cells was little affected by OH-TAM. High concentrations of OH-TAM are required to obtain significant growth inhibition (10^–6^ M, Table 1). As expected, the anti-oestrogen-resistant cell lines, MCF-7LCC1 and MCF-7LY2, appeared less responsive to OH-TAM regarding cell proliferation compared with MCF-7 or MCF-7 7AZ (Table 1).

In addition, the effect of SR31747A on cell proliferation was analysed on NBECs in primary culture. The results illustrated in Figure 1C demonstrated that NBECs were less sensitive than the tumoural cell lines to the growth-inhibitory activity of SR31747A, since a 50% cell growth reduction required 5×10^–7^ M SR31747A (Table 1).

### Effect of SR31747A on the proliferation of human prostatic cancer cell lines

**Figure 2:**
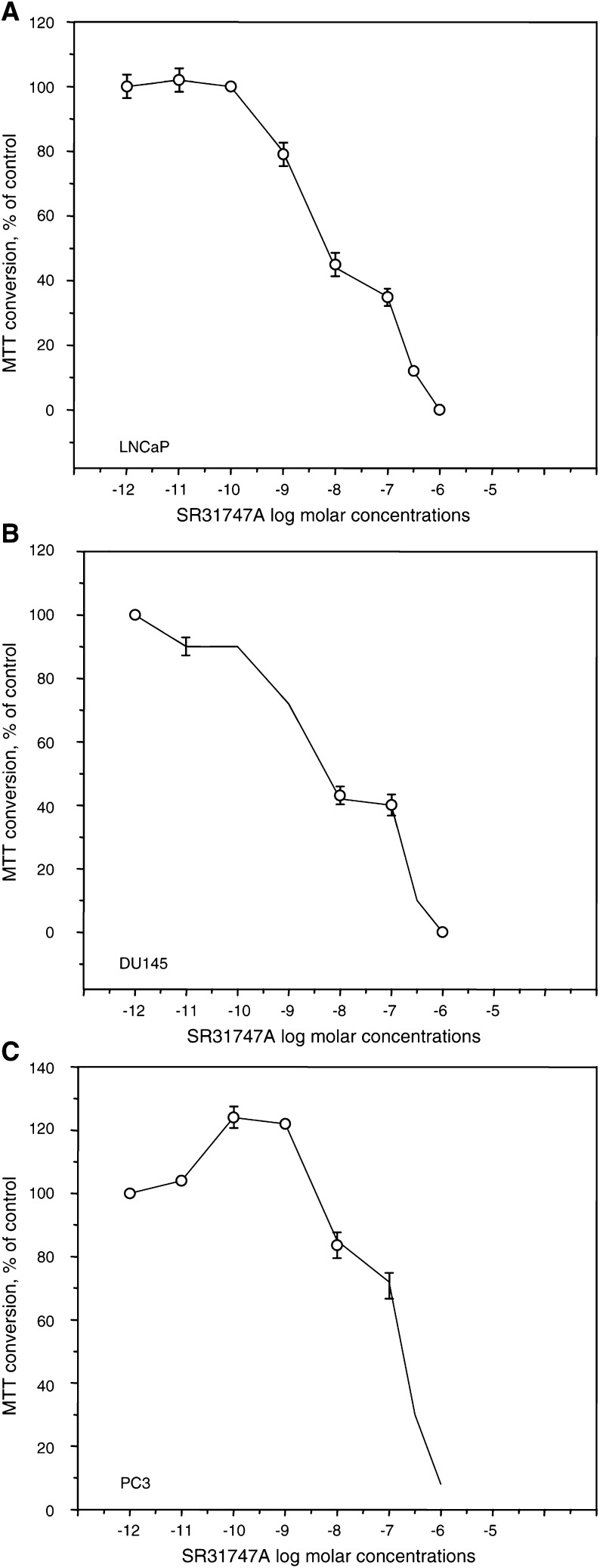
Antiproliferative effect of SR31747A on prostatic cancer epithelial cell lines. Treatments with SR31747A were performed in low lipid serum concentrations (1% FBS), as described in Materials and methods. Effects on hormono-responsive LNCaP cells (**A**), and on hormono-unresponsive DU145 (**B**) and PC3 (**C**) cells. The results are expressed as a percentage of MTT conversion measured in untreated cells.

The antiproliferative activity of SR31747A was further tested *in vitro* on three different human prostatic cancer cell lines. The results presented in Figure 2

and Table 1 indicate that, similar to breast cancer cells, the growth of prostatic cell lines was inhibited by SR31747A *in vitro*. Moreover, the growth inhibitory activity of SR31747A appeared to be dose dependent, and 50% growth inhibition of LNCaP and DU145 cells was obtained with 10^–8^ M SR31747A (Table 1). PC3 cells appeared to be less sensitive than the two other tested prostatic cell lines, in that 50% inhibition was obtained with 10^–7^ M SR31747A. The effect of the antiandrogen flutamide on cell proliferation was compared with that of SR31747A. Interestingly, SR31747A appeared to be more efficient than flutamide in inhibiting the proliferation of the hormono-sensitive LNCaP cells: a 100-fold higher flutamide concentration was required to obtain a similar inhibitory effect (Table 1). As expected and in contrast with LNCaP, the proliferation of the two hormono-unresponsive DU145 and PC3 cell lines was not affected by the flutamide treatment regardless of the concentration tested. The IC_50_ of flutamide on LNCaP lines is 10^–6^ M, in accordance with previous reports (Evans *et al*, 2001; Mitchell *et al*, 2002).

### Analysis of the role of each receptor in mediating SR31747A antiproliferative activity

#### SR31747-induced proliferation inhibition is predominantly mediated by EBP

**Figure 3:**
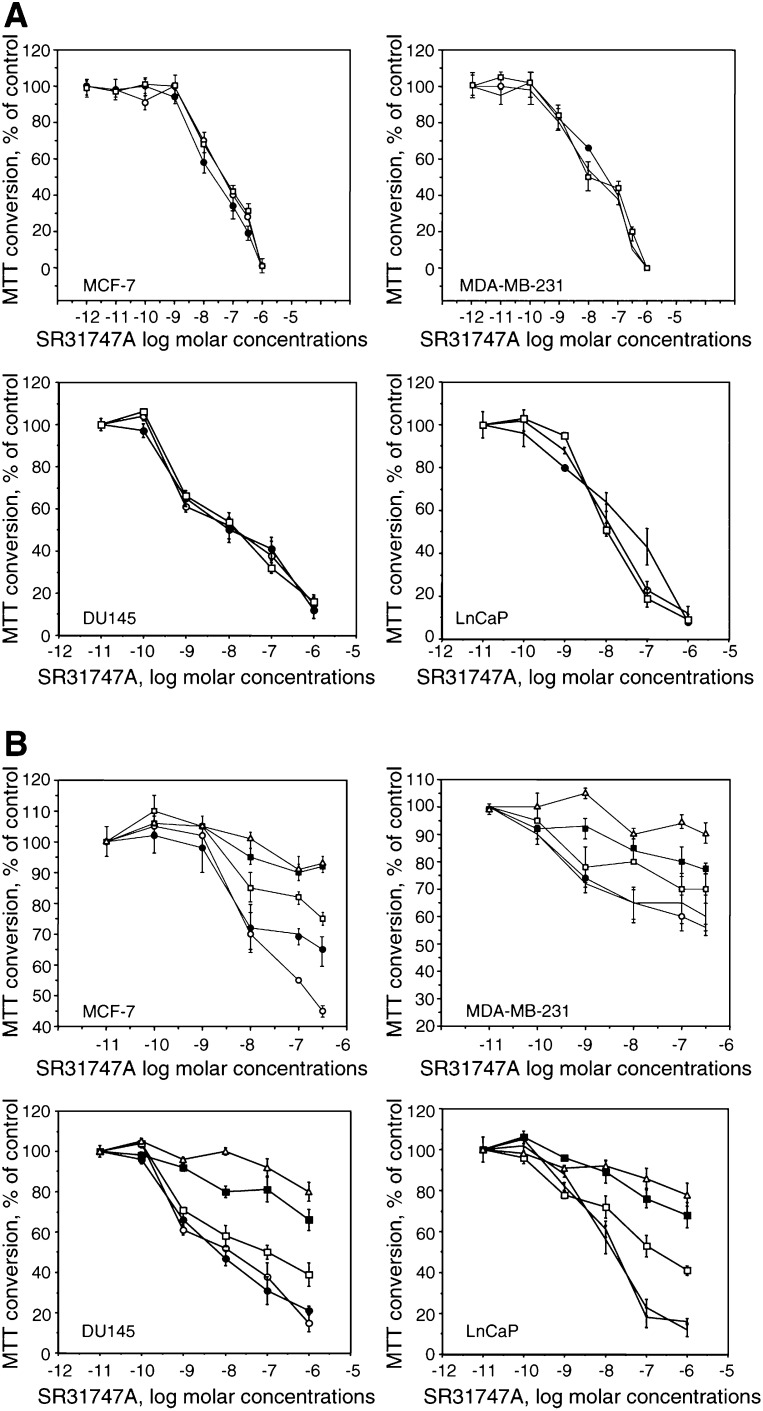
Effect of pentazocine (**A**) or cholesterol (**B**) on the inhibition of mitogenesis in different cell lines induced by SR31747A. (**A**) Two concentrations of (+) pentazocine were tested: control (○), 10^−6^ M (•) and 10^−5^ M (□). (**B**) Four cholesterol concentrations were tested: control (○), 1 *μ*g ml^−1^ (•), 2 *μ*g ml^−1^ (□), 10 *μ*g ml^−1^ (▪) and 20 *μ*g ml^−1^ (△). Treatments were performed in low lipid serum concentrations (0.1% FBS for breast cancer cell lines or 1% for prostate cell lines). Representative curves are shown: MCF7, MDA-MB-231 for breast cancer cell lines, and DU145 and PC3 for prostate cancer cell lines. The results are expressed as a percentage of MTT conversion measured in untreated cells.

We tested whether the inhibition of cell proliferation is mediated by either SR-BP or EBP. Firstly, to discriminate between the two proteins, we used the sigma 1 ligand (+) pentazocine that binds to SR-BP, but does not bind EBP (Jbilo *et al*, 1997). As shown in Figure 3

A, the addition of (+) pentazocine did not affect the antiproliferative activity of SR31747A, even when 10^–5^ M (+) pentazocine was added. Representative curves are shown from breast cancer cell lines, MCF7 and MDA-MB231 cells, and from prostate cancer cell lines, DU-145 and LnCaP (Figure 3A). (+) Pentazocine alone does not affect cell proliferation (data not shown). Secondly, if SR31747A-induced proliferation arrest is because of EBP inhibition, then this effect may be reversed when adding cholesterol. The involvement of EBP was demonstrated as the addition of cholesterol was shown to concentration-dependently counteract the SR31747A antiproliferative effect. Representative curves are shown from breast cancer cell lines, MCF7 and MDA MB 231 cells, and from prostate cancer cell lines, DU-145 and LnCaP (Figure 3B). The antiproliferative effect of SR31747A was partially reduced by adding cholesterol, even at the maximum concentration tested: 20 *μ*g ml^−1^ cholesterol restored cellular proliferation by 90%. Higher percentages were not achieved when using higher cholesterol concentrations (data not shown). Cholesterol itself, in the absence of SR31747, has no effect (data not shown).

#### Determination of EBP and SR-BP expression in human breast and prostate cancer epithelial cells

**Figure 4:**
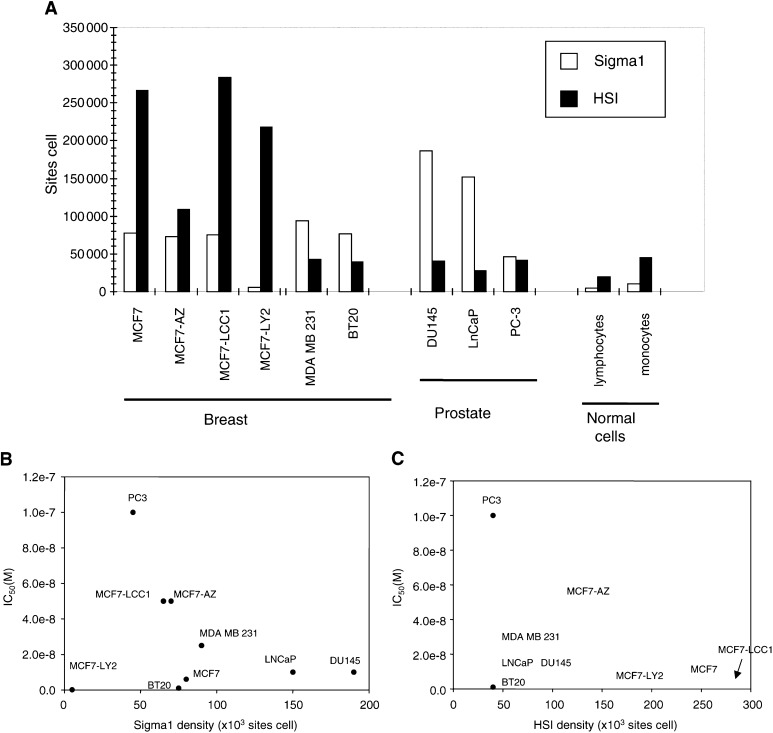
Measurement of EBP and SRBP1 sites in breast or prostatic cancer epithelial cell lines grown in 0.1 or 1% FBS, respectively. (**A**) EBP and SR-BP numbers were determined by flow cytometric analysis of immunofluorescent-stained cells, as described in Material and Methods. For comparative purposes, EBP and SR-BP sites were also evaluated on normal cells, lymphocytes and monocytes. (**B,C**): SR31747A IC_50_ is plotted against SR-BP receptor density (**B**) or EBP density (**C**).

In an attempt to correlate the growth-inhibitory activity of SR31747A with the presence of its receptors, we evaluated EBP and SR-BP expression levels by flow cytometry using specific antibodies in the breast and prostatic cancer cell lines used in this study. Our results reported in Figure 4

A indicate that, except for MCF-7 LY2 cells exhibiting very low levels of SR-BP, all cells examined expressed significant levels of both EBP and SR-BP. Interestingly, significantly higher expression levels in comparison with normal cells were obtained, as quantification of SR-BP and EBP showed receptor densities ranging from 30 000 to 280 000 sites cell^−1^, whereas in normal cells (in lymphocytes or monocytes) EBP or SR-BP density was lower 50 000 sites cell^−1^. Both SR-BP and EBP had expression levels that varied according to the cell line studied. In the breast cancer MCF-7 cell line and MCF-7-derived cell lines, significant amounts of both EBP and SR-BP were obtained from 40 000 to 280 000 and 5 000 to 80 000 sites cell^−1^, respectively. By contrast, BT20 and MDA-MB231 cell lines exhibited higher expression levels of SR-BP than EBP, from 75 000 to 90 000 sites cell^−1^ compared with 40 000 sites cell^−1^, respectively. In prostatic cancer cell lines, 40 000 EBP sites cell^−1^ were measured. Whereas similar SR-BP densities were measured in the PC3 cell line, LNCaP and DU145 were found to express a dramatically greater number of SR-BP (150 000 and 190 000 sites cell^−1^, respectively). To determine whether there is a correlation between the sensitivity to SR31747A treatment and the cellular receptor content, we plotted the SR31747A IC_50_ levels obtained for each cell line (noted in Table 1) against the corresponding SR-BP density (Figure 4B) or EBP density (Figure 4C). No significant correlations were observed. Moreover, there were no significant correlations when considering the EBP/SR-BP content ratio (data not shown).

### Effect of SR31747A on tumour development *in vivo*

To determine whether SR31747A has any antitumoural activity *in vivo*, mammary and prostatic tumoural cell lines were injected s.c. into nude mice on two opposite sites in the flank. SR31747A treatments (25 mg kg^−1^) given daily for 2–3 months did not cause any significant body weight loss relative to controls.

#### Effect of SR31747A on breast tumour development *in vivo*

**Figure 5:**
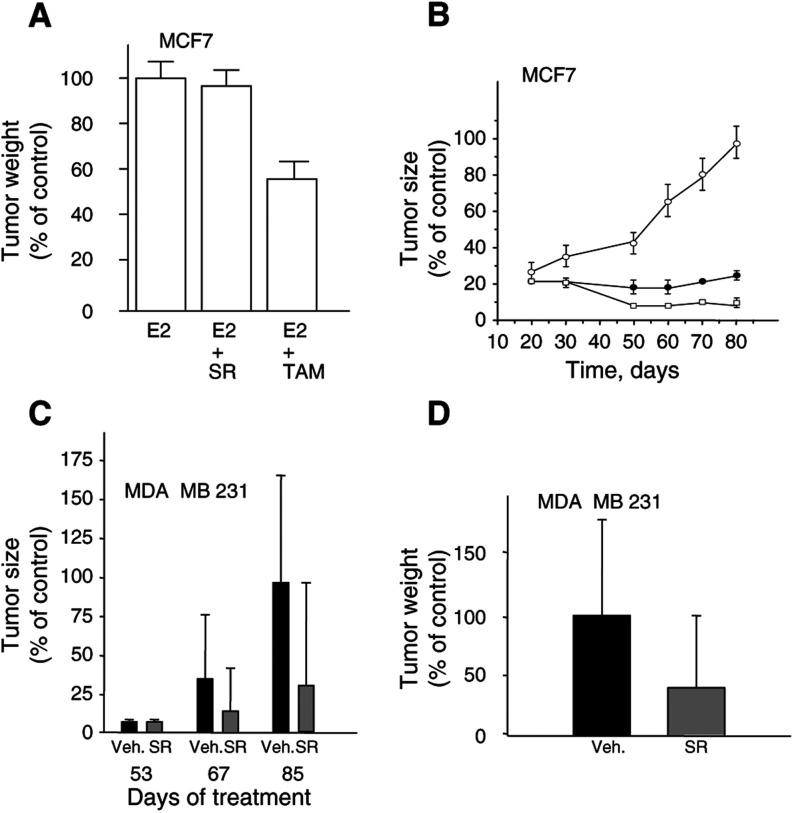
Effect of SR31747A treatment on the development of tumours derived from hormono-responsive MCF-7 cells (**A,B**) or hormono-unresponsive MDA MB 231 (**C,D**) breast cancer cell lines *in vivo*. (**A**) Tumour weight was reported when mice were treated with 2 *μ*g oestradiol (E2) alone or combined with either 25 mg kg^−1^ SR31747A or 1 mg kg^−1^ tamoxifen (TAM). (**B**) SR31747A synergises with tamoxifen to inhibit MCF-7 cell proliferation *in vivo*. Mice bearing MCF-7 cells were treated with 2 *μ*g E2 to induce tumour cell proliferation. These mice were then treated or not (○) with 1 mg kg^−1^ tamoxifen alone (•) or combined with 25 mg kg^−1^ SR31747A (□). Tumour weight and tumour size were plotted as a percentage of tumour weight and size observed in the control group at the end of treatment (day 80). (**C,D**): Mice bearing MDA-MB231 cells were treated with either 25 mg kg^−1^ SR31747 (grey) or vehicle alone (black), as indicated in Materials and Methods. Tumour sizes measured at different treatment times are plotted (**C**) and tumour weight at the end of treatment (day 92) is reported (**D**). Tumour sizes or weights are reported as a percentage of the size or weight of the tumour observed in the control group. Values relative to tumour size are the mean values obtained for each experimental group (total tumour load divided by the number of tumours). Animals per group: 10 and experiments have been performed twice.

First, we tested the impact of SR31747A on tumour development originating from hormono-responsive MCF-7 cells (Figure 5

A). MCF-7 cells formed tumours in only 20% of control animals. When animals were treated daily with E2, more than 90% of mice developed tumours (data not shown). Whereas the antioestrogen TAM partially antagonise the tumoural activity of E2, SR31747A was unable to affect the incidence and size of MCF-7 tumours in E2-treated animal groups (Figure 5A). We investigated the potential synergy between SR31747A and TAM *in vivo* by monitoring the development of MCF7 cell-derived tumours when mice were treated with a combination of both compounds. As mentioned above, all animal groups received daily injection of 2 *μ*g E2 to promote tumour formation and growth. As expected, the tumour size (Figure 5B) appeared to be markedly reduced in the presence of the antioestrogen. However, under 1 mg kg^−1^ TAM treatment, tumours in the vehicule-treated group started to regrow 50–55 days after the beginning of treatment. This process was totally prevented when TAM and SR31747A are associated until the end of treatment (70 days, Figure 5B). Moreover, under these experimental conditions, the addition of SR31747A was even able to induce tumour regression after 30–40 days of treatment (Figure 5B).

While SR31747A alone is not efficient at inhibiting MCF-7 cell-derived tumour growth *in vivo*, the development of tumours that formed after injection of MDA-MB231 cells appeared to be modified substantially under SR31747A treatment. As shown in Figure 5C, SR31747A tended to reduce the growth of tumours derived from the MDA-MB231 cells, by 60% at 67 days and 70% at 85 days compared to control. At the end of treatment (92 days), tumours were 60% lighter in weight in animals treated with SR31747A than in control (Figure 5D).

### Effect of SR31747A on prostatic tumour development *in vivo*

**Figure 6:**
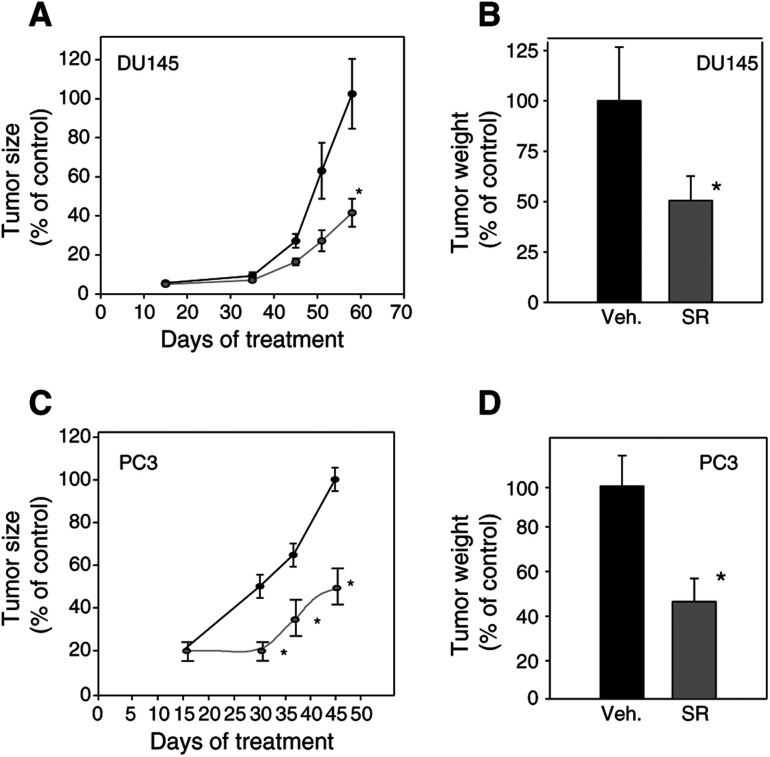
Effect of SR31747A treatment on the development of tumours derived from prostatic hormono-independent DU145 (**A,B**) and PC3 (**C,D**) cancer cells *in vivo*. Mice were treated with either 25 mg kg^−1^ SR31747A (grey) or vehicle alone (black), as indicated in Materials and Methods. Tumour size is plotted as a function of the day of treatment (**A,C**) and tumour weight at the end of treatment is reported (**B**, day 60 for DU145; **D**, day 45 for PC3). Sizes or weights of tumours in each group are reported as a percentage of the size or weight of tumours observed in control at the end of treatment, that is, day 60 and day 45 for DU145 and PC3, respectively. Animals per group: 10 and experiments have been performed twice.

**Figure 7:**
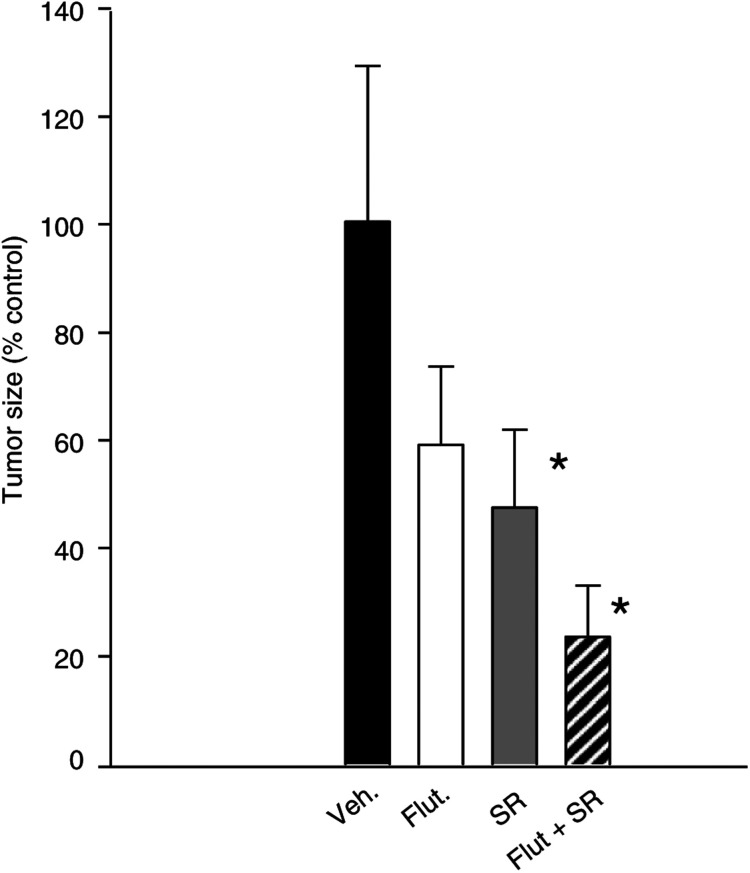
SR31747A synergises with flutamide to inhibit cell proliferation *in vivo*. Mice bearing LNCaP cells were treated with 2 *μ*g testosterone, then with 2.5 mg kg^−1^ flutamide (white) and 25 mg kg^−1^ SR31747A (grey) alone or in combination (black lined). Sizes are reported as a percentage of the size of the tumour observed in control at the end of treatment (day 75). Animals per group: 10 and experiments have been performed twice.

The impact of SR31747A on the development of tumours originating from hormono-independent prostatic carcinomas DU145 and PC3 was tested (Figure 6

). In both cases, tumour development was significantly slowed down. A 25 mg kg^−1^ dose of SR31747A treatment resulted in a reduction of both the tumour size (Figure 6A, C) and tumour weight (Figure 6B, D), that is, by 50 and 40% for PC3 and DU145 tumours, respectively. SR31747A treatment did not affect the body weight. In this model, we also tested a putative synergistic effect of SR31747A with the anti-androgen flutamide. When considering the development of hormono-dependent LNCaP cells, we noted that SR31747A (25 mg kg^−1^) and flutamide (2.5 mg kg^−1^) synergistically inhibited LNCaP cell proliferation *in vivo*. Tumour size was reduced by 45 and 55% following SR31747A or flutamide treatment, respectively (Figure 7

). A combined SR31747A and flutamide treatment resulted in a 70% reduction in tumour size at day 75 (Figure 7).

## DISCUSSION

Previous studies have clearly shown that SR31747A has immunosuppressive properties. Here we further document the *in vitro* antiproliferative effect of SR31747A, and demonstrate for the first time that the molecule also inhibits proliferation *in vivo* in the mouse xenograft model. Our results showed that SR31747A inhibited cell proliferation of either hormono-responsive or hormono-unresponsive human cancer cell lines *in vitro*. We obtained evidence on the nanomolar potency of the SR31747A molecule in inhibiting the proliferation of breast cancer MCF7 cells, MCF-7 derived cells, MDA MB 231 and BT20 cells. This inhibitory effect of SR31747A was also reproduced in both hormono-responsive (LNCaP) and -unresponsive (DU-145 and PC3) prostate cancer cell lines. Moreover, *in vivo*, using the mouse xenograft model, SR31747A treatment was shown to reduce significantly tumour development when induced by the inoculation of human breast or prostatic cancer cell lines in nude mice. No toxicity was noted as the animal body weight was not affected during treatment. Interestingly, we also found that SR31747A: (i) prevents the escape of breast cancer cells under long-term tamoxifen treatment and (ii) synergises with flutamide.

The nanomolar efficacy of SR31747A in inhibiting cell proliferation *in vitro* is in favour of a receptor-mediated event. In humans, SR31747A is known to bind two proteins. In this context, it is crucial to identify which protein mediates the antiproliferative activity of the molecule in mammals. To explore SR-BP and EBP involvement, the antiproliferative effect of SR31747A was challenged with either (+) pentazocine, an SR-BP-specific ligand, or cholesterol. The inhibitory effect of SR31747A was not antagonised by the addition of the competitive SR-BP ligand (+) pentazocine, whereas the addition of cholesterol was found to reduce partially and dose dependently the SR31747A inhibitory effect. These data are in accordance with previous results (Labit-Le Bouteiller *et al*, 1998) and support the involvement of EBP in mediating the antiproliferative effect of SR31747A. However, some lines of evidence suggest that EBP may not be the only protein involved. First, the antiproliferative effect of SR31747A was partially prevented by cholesterol. Second, if the antiproliferative effect of SR31747A is exclusively a consequence of the sterol isomerase inhibition, then the sensitivity to SR31747A may be correlated with the cellular EBP content. To test this hypothesis, we investigated the expression levels of both SR-BP and EBP in the series of cancer cell lines included in our study. We did not obtain a significant correlation between the IC_50_ and the EBP content. Cell lines that express markedly different levels of EBP, such as MCF-7-AZ and LNCaP or DU145, were found to exhibit sensitivity similar to SR31747A. The inverse was also observed. Similar results were obtained with SR-BP. Two different hypotheses may be put forward to explain this phenomenon. First, when analysing EBP and SR-BP subcellular expression, both proteins were previously shown to be colocalised at the endoplasmic reticulum and nuclear envelope (9), thus indicating that EBP and SR-BP may be mutually involved in mediating SR31747A antiproliferative activity. However, no physical interaction between both proteins has been observed so far, and our data indicate that the cellular sensitivity to SR31747A does not depend on either EBP or SR-BP levels or any combination of these two proteins. Second, the possibility that additional binding sites for SR31747A may exist in humans cannot be overlooked. Interestingly, preliminary binding studies have shown that 45 nM SR31747A displaced ^3^H DTG binding to sigma2 (H Vidal, personal communication). Sigma2 receptors are proteins of approximately 21 kDa whose sequence is unknown. To date, the protein properties have been investigated using specific pharmacological tools. Although its function remains elusive, some reports suggest that it may play a role in tumourigenesis. Using specific ligands that can discriminate between SR-BP and sigma2, sigma2 receptors were found to be overexpressed in tumour cells as compared with their normal counterparts, including breast, neural, lung, prostate and melanoma tumours (Vilner *et al*, 1995). In addition, sigma2 receptor expression was found to be correlated with the proliferative status of human breast tumours (Mach *et al*, 1997) and solid tumours (Wheeler *et al*, 2000). It has thus been demonstrated that sigma2 receptor binding ligands may be used for *in vivo* noninvasive diagnostic receptor imaging (John *et al*, 1999). In this context, it would be essential to identify the sigma2 protein. Further studies are needed to investigate the biological responses associated with SR31747A binding on sigma2 and to define accurately the SR31747A mode of action. It would therefore be very useful to develop cell lines lacking one or several SR31747A receptors. MCF-7-LY2 cells that lack detectable levels of SR-BP could be of interest.

In conclusion, we report for the first time that, in addition to its immunoregulatory property, SR31747A exhibits antiproliferative activity both *in vitro* and *in vivo* on a series of human breast and prostate cancer cell lines. In order to document accurately SR31747A antitumoural activity, further studies are now required to decipher its mode of action, to identify accurately the sigma2 protein, and to define the separate roles of each SR-BP.
